# Cardiac BNP gene delivery prolongs survival in aged spontaneously hypertensive rats with overt hypertensive heart disease

**DOI:** 10.18632/aging.100655

**Published:** 2014-05-03

**Authors:** Jason M. Tonne, Sara J. Holditch, Elise A Oehler, Claire A. Schreiber, Yasuhiro Ikeda, Alessandro Cataliotti

**Affiliations:** ^1^Department of Molecular Medicine, Mayo Clinic, College of Medicine, Rochester, MN 55905, USA; ^2^Cardiorenal Research Laboratory, Division of Cardiovascular Diseases, Departments of Medicine and Physiology, Mayo Clinic, College of Medicine, Rochester, MN 55905, USA

**Keywords:** natriuretic peptide, BNP, hypertension, survival, AAV9

## Abstract

**Background:**

Hypertension is a highly prevalent disease associated with cardiovascular morbidity and mortality. Recent studies suggest that patients with hypertension also have a deficiency of certain cardiac peptides. Previously we demonstrated that a single intravenous injection of the myocardium-tropic adeno-associated virus (AAV) 9-based vector encoding for proBNP prevented the development of hypertensive heart disease (HHD) in spontaneously hypertensive rats (SHRs). The current study was designed to determine the duration of cardiac transduction after a single AAV9 injection and to determine whether cardiac BNP overexpression can delay the progression of previously established HHD, and improve survival in aged SHRs with overt HHD.

**Methods and Results:**

To evaluate the duration of cardiac transduction induced by the AAV9 vector, we used four week old SHRs. Effective long-term selective cardiac transduction was determined by luciferase expression. A single intravenous administration of a luciferase-expressing AAV9 vector resulted in efficient cardiac gene delivery for up to 18-months. In aged SHRs (9-months of age), echocardiographic studies demonstrated progression of HHD in untreated controls, while AAV9-BNP vector treatment arrested the deterioration of cardiac function at six months post-injection (15-months of age). Aged SHRs with established overt HHD were further monitored to investigate survival. A single intravenous injection of the AAV9-vector encoding rat proBNP was associated with significantly prolonged survival in the treated SHRs (613±38 days, up to 669 days) compared to the untreated rats (480±69 days, up to 545 days)(p<0.05).

**Conclusions:**

A single intravenous injection of AAV9 vector elicited prolonged cardiac transduction (up to 18 months post-injection). AAV9 induced cardiac BNP overexpression prevented development of congestive heart failure, and significantly prolonged the survival of aged SHRs with previously established overt HHD. These findings support the beneficial effects of chronic supplementation of BNP in a frequent and highly morbid condition such as HHD.

## INTRODUCTION

Hypertension (HTN) is a major contributor to the global burden of cardiovascular disease, leading to stroke, myocardial infarction, heart failure (HF), and death [1]. The myocardial complications result from increased mechanical load on the heart, which eventually leads to overt hypertensive heart disease (HHD), characterized by diastolic dysfunction, cardiac remodeling and fibrosis. Under physiological conditions of increased myocardial load and myocardial stretch, the heart synthesizes and secretes two peptide hormones – atrial natriuretic peptide (ANP) and B-type natriuretic peptide (BNP) – that are endogenous ligands for the particulate guanylyl cyclase receptor A (NPR-A) [2-4]. ANP and BNP synthesis and secretion contribute to the maintenance of optimal cardiorenal and blood pressure homeostasis. Following receptor binding and generation of the second messenger 3', 5'- cyclic guanylyl monophosphate (cGMP), the natriuretic peptides (NPs) mediate biological actions which include natriuresis, inhibition of the renin-angiotensin-aldosterone system, and vasodilatation. There are also important local autocrine and paracrine actions of the NPs in the heart such as inhibition of fibrosis, hypertrophy, and enhancement of diastolic function [5].

Studies indicate that in subjects with cardiovascular diseases the biological structure of these hormones may be altered, thus reducing their protective activities [6, 7]. Furthermore, recent studies have established that basal blood pressure and risk for HTN are linked to common genetic variants of the ANP (*NPPA*) and BNP (*NPPB*) genes [8]. These genetic variants are associated with reduced circulating levels of ANP and BNP and are characterized by elevated blood pressure and an increased risk for HTN. Consistent with Newton-Cheh's study, we demonstrated a lack of activation of the BNP system (BNP1-32 and NT-proBNP) in grade 1 HTN [9]. Recently, we have completed a study in a population from Olmsted County, MN, that confirmed and extended this observation to the general population, where circulating forms of ANP were also reduced in subjects with elevated blood pressure [10, 11]. All together these findings indicate that HTN is associated with a derangement of the natriuretic peptide system which is characterized by the lack of activation of biologically active cardiac NPs.

These findings led us to investigate the effect of prolonged cardiac overexpression of BNP in a model of progressive HHD [12]. Here, using the myocardium-tropic adeno-associated virus 9 (AAV9)-based vector, we examined the effect of long-term (up to nine months) rat proBNP expression in spontaneously hypertensive rats (SHRs). Specifically, after a single AAV9 intravenous injection in four week-old SHRs, BNP overexpression prevented the development of HHD for up to nine months. The duration of this overexpression, after a single injection, is unclear. Furthermore, it remains to be determined whether cardiac overexpression of BNP can reverse overt HHD, through enhancement of cardiac function and structure, and improve survival in older SHRs where overt HHD is already established.

The current study was therefore designed to determine whether a single intravenous injection of AAV9 is associated with prolonged cardiac selective overexpression of BNP and whether cardiac BNP overexpression induced in the setting of overt HHD, could enhance cardiac function and structure or delay progression of HHD and improve survival in aged (nine months) SHRs. We hypothesized that AAV9 elicits long-term specific cardiac transduction and that cardiac BNP overexpression, compared with untreated rats, can improve cardiac function and structure, and survival even when HHD is already established in aged SHRs.

## RESULTS

### Systemic AAV9 administration facilitates long-term cardio-specific gene transduction in young SHRs

**Figure 1:**
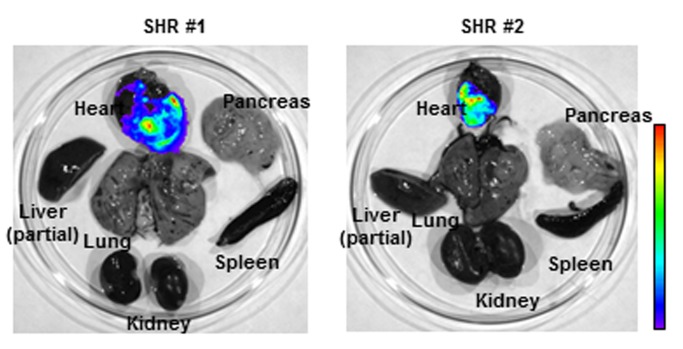
AAV9 vector facilitates long-term cardiac gene delivery in spontaneously hypertensive rats (SHR). Distribution of luciferase activities in SHR organs was monitored by Xenogen IVIS Living Image 18 months after systemic adeno-associated virus (AAV) vector administration. Strong luciferase expression in heart demonstrated efficient long-term cardiac gene delivery by AAV9 vector in SHR.

Four week-old SHRs were intravenously transduced by the luciferase-expressing AAV9 vector at a dose of 10^13^ genome copies per kg. IVIS luciferase imaging revealed long-term cardiac luciferase expression at 18 months after single AAV9 intravenous administration (Fig. 1). Importantly, no strong luciferase signal was observed in other harvested organs, such as liver, kidney and lung.

### AAV9 vector-mediated cardiac proBNP over-expression prevents the progression of hypertensive heart disease

Nine month-old SHRs, characterized by overt HHD with reduced diastolic and systolic function and altered cardiac structure [12], were randomly assigned to placebo (control rats) or treated with the AAV9 vector encoding rat proBNP at a dose of 10^13^ gc/kg (Table 1).

**Table 1:** Echocardiographic parameters in untreated and BNP-treated SHRs

Cardiac parameters	Pre-AAV9 (9m, n=6)	Pre-Control (9m, n=6)	AAV9-proBNP (15m, n=6)	Control (15m, n=6)
HR	377±23	391±10	381±19	361±26
IVSd (mm)	2.58±0.13	2.40±0.14	2.29±0.20*	
LVIDd (mm)	7.71±0.05	7.64±0.34	7.81±0.78	8.59±0.94**
LVPWd (mm)	2.23±0.19	2.12±0.13	2.26±0.23	2.30±0.14**
IVSs (mm)	3.43±0.03	3.41±0.21	3.35±0.19	3.92±0.36**#
LVIDs (mm)	4.94±0.69	4.73±0.13	4.69±0.56	5.79±1.34
LVPWs (mm)	3.29±0.30	3.22±0.20	3.43±0.33	2.99±0.34 #
LVMd (g)	1.88±0.22	1.73±0.13	1.77±0.14	2.15±0.39**#
EF (%)	72.2±3.8	74.8±1.8	76.2±3.3	66.5±11.5
%FS	36.3±4.5	38.8±1.6	40.0±3.0	33.3±7.4

Adeno associated virus serotype 9, AAV9; month, m; heart rate, HR; Interventricular Septum diastole, IVSd; Left Ventricular Internal Dimension diastole, LVIDd; Left Ventricular Posterior Wall diastole, LVPWd; Interventricular Septum systole, IVSs; Left Ventricular Internal Dimension systole, LVIDs; Left Ventricular Posterior Wall systole, LVPWs; Ejection Fraction, EF; Percentage Fraction Shortening, %FS; Left Ventricular Mass diastole, LVMd.

* Pre-AAV vs. AAV9 proBNP treated at 15m (p<0.05);

** Pre-Control vs. Control at 15m (p<0.05);

# Control at 15m vs. AAV9 proBNP treated at 15m (p<0.05)

Echocardiographic analysis of the cardiac parameters of control rats at 9 and 15 months revealed progression of HHD upon aging (Table 1). In contrast, SHRs with AAV9 vector-mediated proBNP over-expression showed limited changes in cardiac parameters at 6 months post vector administration, suggesting prevention of HHD progression by proBNP over-expression in aged rats. Indeed, when compared with untreated rats, treated SHRs showed significantly better systolic and diastolic function 6 months post AAV9 injections and no signs of deterioration of any echocardiographic parameters assessed before treatment (Table 1).

**Figure 2:**
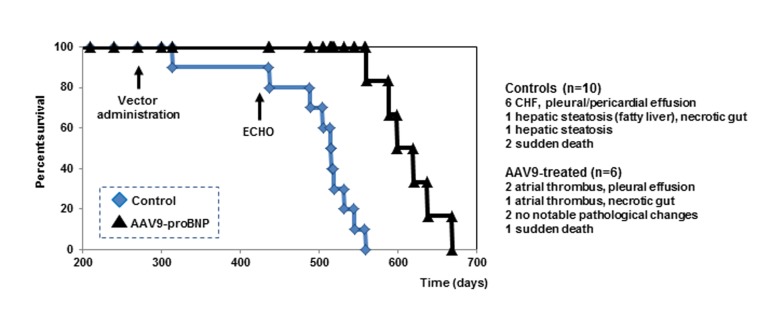
Cumulative survival curves Kaplan-Meier survival curve of untreated controls (n=10, blue squares) (life span from 314 to 545 days) and treated rats (n=6, black triangles) (life span from 589 to 638).

Treated and untreated SHRs were further monitored for survival. AAV9-BNP vector treatment significantly improved survival in aged SHRs with previously developed HHD (Fig. 2). A single intravenous injection of the AAV9-vector significantly prolonged the survival of treated SHRs (613±38 days), compared to the untreated rats (480±69 days)(p<0.05).

**Figure 3:**
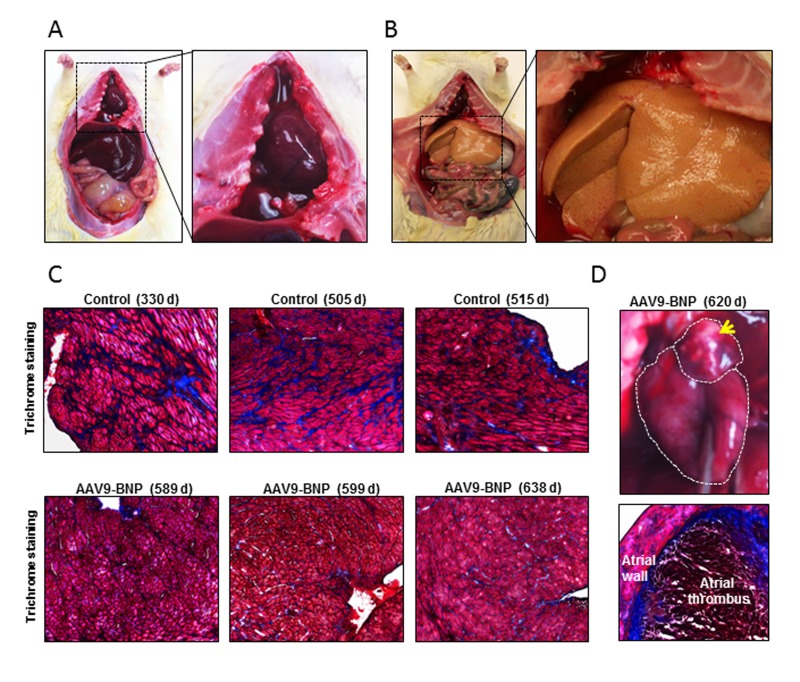
Pathological assessment after long-term proBNP over-expression in aged spontaneously hypertensive rats (SHRs) with established HHD The major clinical signs found in euthanized rats are listed. (**A**) A representative image of a SHR with pleural effusion. Note the chest cavity filled with clear liquid (right panel). (**B**) A representative image of a control SHR demonstrating hepatic steatosis. A higher magnification of the fatty yellow liver is shown in a right panel. (**C**) Mason's Trichrome staining of heart sections of controls and adeno-associated virus (AAV) vector-treated rats. Note prominent connective tissue deposits (stained in blue) in control rats, even though those control rats were younger than AAV9-treated rats. (**D**) A representative image of atrial thrombus (upper panel) and trichrome staining of the atrial thrombus (lower panel).

Among 10 untreated SHRs, 6 were found lethargic on days 314, 505, 515, 519, 532 and 545, with labored breathing and no appetite. Those rats were euthanized and tissues were harvested for pathological testing. Pathological changes typical of congestive HF including pericardiac and pleural effusion (6 out of 6) and ascites (2 out of 6) were observed in those rats (Fig. 3A). Two other control rats were found lethargic with black feces and dark urine on days 437 and 515, and were euthanized. They demonstrated notable hepatic steatosis (yellow fatty liver, Fig. 3B) and necrotic gut (1 out of 2). Two other control rats suddenly died over weekend (days 437 and 489) without showing any notable clinical symptoms. Echocardiographic examinations indicated that systolic dysfunction and HF were a frequent cause of death among the untreated rats (e.g. EF ~40% the day before death for an untreated rat).

Three out of 6 AAV9-proBNP-treated rats became lethargic at 589, 620 and 638 days of age and were euthanized. Pathological studies demonstrated notable left atrial thrombosis in their hearts (3 out of 3; Fig. 3D). The 620- and 638-day-old rats also showed pleural effusion. Two AAV treated rats became lethargic and euthanized on days 599 and 638. No notable pathological change was observed in those rats. One treated rat suddenly died over-weekend (day 560) and no tissue was available for pathology.

When cardiac tissues were assessed for cardiac fibrosis, a trend of increased connective tissue areas, which were stained blue by Mason's trichrome staining, was observed (Fig. 3C).

Importantly, untreated SHRs had a significant increase in blood pressure at 15 months as compared with pre-randomization period, while BNP-treated SHRs did not have change in blood pressure over time. Of note, cardiac BNP overexpression was not associated with a significant reduction in blood pressure, yet a tendency to a lower systolic and diastolic pressure was observed in the treated SHRs as compared with untreated rats 6 months after vector administration (Table 2).

**Table 2:** Systolic, diastolic, and mean blood pressure in untreated (Control) and AAV9-BNP vector-treated SHRs

	Pre (day -2, 9m)		Day 4 (9m)		Day 56 (11m)		Day 186 (15m)	
BNP	Control	+AAV9-BNP	Control	+AAV9-BNP	Control	+AAV9-BNP	Control	+AAV9-BNP
Systolic	201±16	199±22	203±12	192±15	220±13	202±24	221±6*	205±24
Diastolic	145±18	147±22	142±12	142±13	168±5	160±32	177±10*	162±32
Mean	164±17	164±22	162±12	159±14	185±8	172±27	187±8*	176±30

* Pre-Control vs. Control at 15m (p<0.05)

## DISCUSSION

We previously reported that a single intravenous injection of AAV9-vector elicited long-term cardiac BNP overexpression, leading to the prevention of HHD in SHRs [12]. However, it was unclear whether cardiac BNP overexpression could reverse or delay progression of established HHD, with both cardiac dysfunction and remodeling, and whether it could improve survival when HHD is fully developed. Therefore, in the current study we extended our previous work to a clinically relevant model of established HHD to investigate whether a single intravenous injection of AAV9-BNP could ameliorate the cardiac dysfunction present in aged SHRs or prevent further worsening and extend survival. Indeed, our findings indicated that cardiac BNP overexpression, induced by a single intravenous injection of AAV9 vector in nine month old SHRs with established HHD, improved both cardiac function and structure as compared to control and delayed the progression to HF. Furthermore, cardiac BNP overexpression significantly extended the life span of aged SHRs with pre-existing HHD.

It is known that the most potent vasodilating cardiac hormone is ANP, however, BNP has more pronounced anti-fibrotic, anti-hypertrophic, and pro-lusitropic properties that may have contributed to the favorable cardiac effects observed here. Moreover, we have previously demonstrated that the favorable effects of cardiac overexpression are, in part, independent of blood pressure reduction [12] and that the cardio-protective effect is rather the results of well-established pleiotropic actions of BNP [13-16]. In this study, we found blood pressure of control SHRs significantly increased overtime; the untreated SHRs had an increase in systolic blood pressure of approximately 20 mmHg from 9 months (pre-randomization) to 15 months of age. Similar increases were observed for diastolic and mean arterial pressure (32 and 23 mmHg, respectively). In contrast, BNP-treated SHRs had only a 6 mmHg increase in systolic blood pressure throughout the study, and the rats were protected from significant increases in BP for 6 months. Those observations indicate modest BP lowering effects of the AAV9-BNP vector in HHD. Thus, this rather subtle increase in blood pressure, compared to the more pronounced elevation in blood pressure in the control rats, may be responsible for the extended survival in the BNP-transfected SHR, as even a few mmHg of reduction/increase are associated with a reduction/increase in risk for cardiovascular events in humans [17].

Although this study has not been designed to investigate the cause of aging, a new shift in the aging process paradigm, so called “hyperfunction theory”, has recently been proposed [18-20], and it might help in the understanding of the favorable survival effects observed with BNP. Indeed, hypertensive heart disease and hypertrophy are good examples of aging-associated hyperfunction also dependent on mTOR pathway [21]. To further confirm this theory, rapamycin has been shown to be effective in reducing cardiac hypertrophy in rodents [22, 23]. Furthermore, relevant to our study, a recent investigation has also shown that activation of the mTOR pathway is associated with increased gene expression of BNP and ANP, while its suppression with rapamycin is associated with a reduced expression of NPs gene [24]. It is, therefore, possible that the anti-hypertrophic anti-fibrotic effects of BNP are independent of the mTOR pathway. A combined use of rapamycin with BNP or other cardiac hormones may further enhance the cardiac anti-remodeling effects observed here thus further extending survival. Further studies are warranted to investigate the possible additive effect of rapamycin and BNP supplementation in experimental HHD.

In humans, hepatic steatosis is frequently associated with hypertension and to the underlying metabolic disease common in many hypertensives (i.e. insulin resistance). It is also known that vascular production of reactive oxygen species (ROS) is increased in hypertension and this may further contribute to the pathogenesis of hepatic steatosis. Similarly, in the current study, SHRs spontaneously developed hepatic steatosis as previously reported by others [25]. In contrast, BNP-transfected SHRs did not develop hepatic steatosis. The lack of hepatic steatosis in the treated rats was an unexpected finding; therefore, we did not control for insulin or other markers of metabolic syndrome, such as lipid profile (i.e. HDL cholesterol, triglycerides, remnant lipoproteins, etc.) and/or ROS. It is possible that BNP has reduced insulin resistance, improved dyslipidemia, and/or reduced ROS production in the treated rats, as these actions have been previous described in both humans and experimental studies [26-33]. Further in-depth studies are warranted to investigate the underlying mechanistic actions that drive the reduced steatosis observed in the BNP-treated rats.

Some treated rats with extended survival, however, demonstrated extensive left atrial thrombosis, which likely was the main cause of death. A major cause of atrial thrombosis in humans is atrial fibrillation (AF). BNP is often elevated in AF due to the increased atrial stretch, but it has never been demonstrated its role in inducing AF. In contrast to normally circulating forms of NPs, a mutant form of ANP has been associated with familiar AF [34]. Although the causal relationship between BNP and thrombogenesis remains unknown, studies have reported that high levels of circulating immuno-reactive BNP is associated with increased risk of thromboembolic events [35, 36]. Further studies are needed to investigate whether the observed atrial thrombosis was induced by BNP overexpression or it was primarily related to aging. Indeed, it is well established that age is a main risk factor for both AF and thrombosis [37] and our BNP-treated rats far exceeded life span of all their untreated littermates reaching a significantly older age than controls.

AAV is a non-pathogenic, single-stranded DNA virus that belongs to the family *Parvoviridae*. Multiple naturally occurring serotypes of AAV exhibit unique receptor usage and tissue tropisms [38, 39]. AAV-based vectors have emerged as promising gene delivery vehicles because of their low toxicity, efficient gene delivery into non-dividing cells and ability to persist long term in vivo [40, 41]. Indeed, recent phase I and phase II clinical trials using AAV vectors have established their safety, in some cases with clinical benefits, in patients with hemophilia B, congenital degenerative eye disease or muscular dystrophy, which paved the path to AAV vector gene therapies for various human disease conditions [42-44]. In cardiac gene therapy, intracoronary injections of AAV1 vector has been used to enable cardiac overexpression of SERCA2a in humans with HF with no notable toxicity [45, 46]. Others and we have demonstrated efficient cardiac gene transduction by a naturally cardiotropic AAV9 vectors upon systemic vector delivery [12, 47, 48], and AAV9 vector is a highly promising vector to achieve cardiac overexpression. The use of systemic administration of AAV9 vectors is safe and easy to perform. Therefore, it has potential applicability in patients that do not require cardiac catheterization or for patients at high risk for contrast induced acute renal failure such as patients with end stage renal diseases and concomitant resistant hypertension with HHD. Indeed, we have recently demonstrated that the use of subcutaneous BNP in a patient with uncontrolled hypertension, despite multiple optimal anti-hypertensive medications, could normalize blood pressure without further medication for over 36 hours [49]. This observation supports the potential beneficial effects of chronic BNP supplementation in uncontrolled hypertension that is often associated with cardiac remodeling and dysfunction (i.e. HHD). Furthermore, since cardiac BNP overexpression achieved via AAV9 vector delivery not only contributes to reduce blood pressure when started early [12], it also improves cardiac function, structure and even survival, as reported in the current study even if BNP over-expression is started after the onset of HHD. Therefore, cardiac BNP gene delivery could provide a new platform to treating high risk subjects such as resistant hypertensive patients with concomitant HHD, leading to the prevention or delay of the development of cardiac dysfunction and remodeling which, together with high blood pressure, are responsible for the high risk of cardiovascular events [50].

In conclusion, in the current study, a single intravenous injection of AAV9 elicited sustained BNP induced-favorable effects on cardiac function and structure with significant, but modest, blood pressure lowering effects. Previously, a significant drop in blood pressure was observed when overexpression of BNP was induced at an earlier time [12], suggesting that early intervention with BNP supplementation may have even further beneficial effects upon cardiac structure, function, and survival. Importantly, better cardiac function and structure was associated with significantly extended survival in these aged SHRs with pre-established HHD. Given that a single AAV9 injection elicited very long cardiac transduction (at least up to 18 months, Figure 1), cardiac gene delivery of BNP could be useful in subjects with uncontrolled hypertension and concomitant HHD to improve cardiac function and structure. The extended survival observed in the current study also suggests that BNP-mediated cardio-protection can reduce the risk of cardiovascular events. The current investigation extended our previous findings where a single intravenous injection of AAV9 elicited sustained cardiac BNP overexpression in juvenile SHRs, persistent reduction in blood pressure and prevention of the development of HHD with age. Here, in a more clinically relevant model with established HHD in aged SHRs, a single intravenous injection of AAV9, not only prevented worsening of cardiac function and structure, but also significantly extended survival of treated old SHRs, compared with untreated controls. Further studies are warranted to investigate the mechanistic effects that underlie the improved survival achieved by a single systemic injection of AAV9-induced cardiac transfection of BNP.

## METHODS

### Animals

Four week-old and 8 month-old SHRs were purchased from Charles River. Sixteen aged male SHRs, 9 months old at the time of vector/placebo (i.e. saline) administration, with impaired cardiac function were randomly assigned to two groups: 6 rats were intravenously injected with proBNP-expressing AAV9 vector (1 x 10^12^ genome copies/rat) (group 1), while 10 untreated SHRs served as controls (group 2). The effects of sustained proBNP expression on cardiac function/remodeling were analyzed six months post injection. All animal studies were approved by the Institutional Animal Care and Use Committee.

### AAV9 vectors

The luciferase- and rat proBNP-expressing AAV9 vectors were produced in human 293T cells using the helper-free transfection method, and the vector titers (genomic copy numbers/ml) were determin-ed by quantitative PCR as reported previously [12].

### Non-invasive tail blood pressure measurement

The CODA High-Throughput Non-Invasive Tail Blood Pressure System (Kent Scientific) was used to monitor the blood pressure of conscious rats.

### IVIS imaging

A Xenogen IVIS biophotonic imaging machine was employed to monitor cardiac luciferase expression. Upon intraperitoneal administration of luciferin, anesthetized rats were euthanized. The organs were harvested immediately, placed on 10 cm plates in the imaging chamber and a background photo of the tissues and a color overlay of the emitted photon data were obtained.

### Echocardiography for NoninvasiveAssessmentof VentricularFunctionandStructure

To evaluate cardiac function and structure in the BNP vector-treated and untreated SHRs, we performed echocardiography at 1 week prior to vector/placebo administration (pre-randomization) and 6 months post injection. All echocardiography examinations were performed by a skilled sonographer blinded to the treatment.

### Masson Trichrome Staining

Sections of frozen cardiac samples were assessed for collagen content by Trichrome staining as reported previously [12].

### Sample Size and Statistical Analysis

Groups were compared with unpaired *t* tests; changes within groups were assessed by paired *t* tests. Comparisons of blood pressure values between groups were performed by 2-way ANOVA for repeated measurements. Data are expressed as mean ± SD. Results were considered significantly different at a level of p<0.05.
